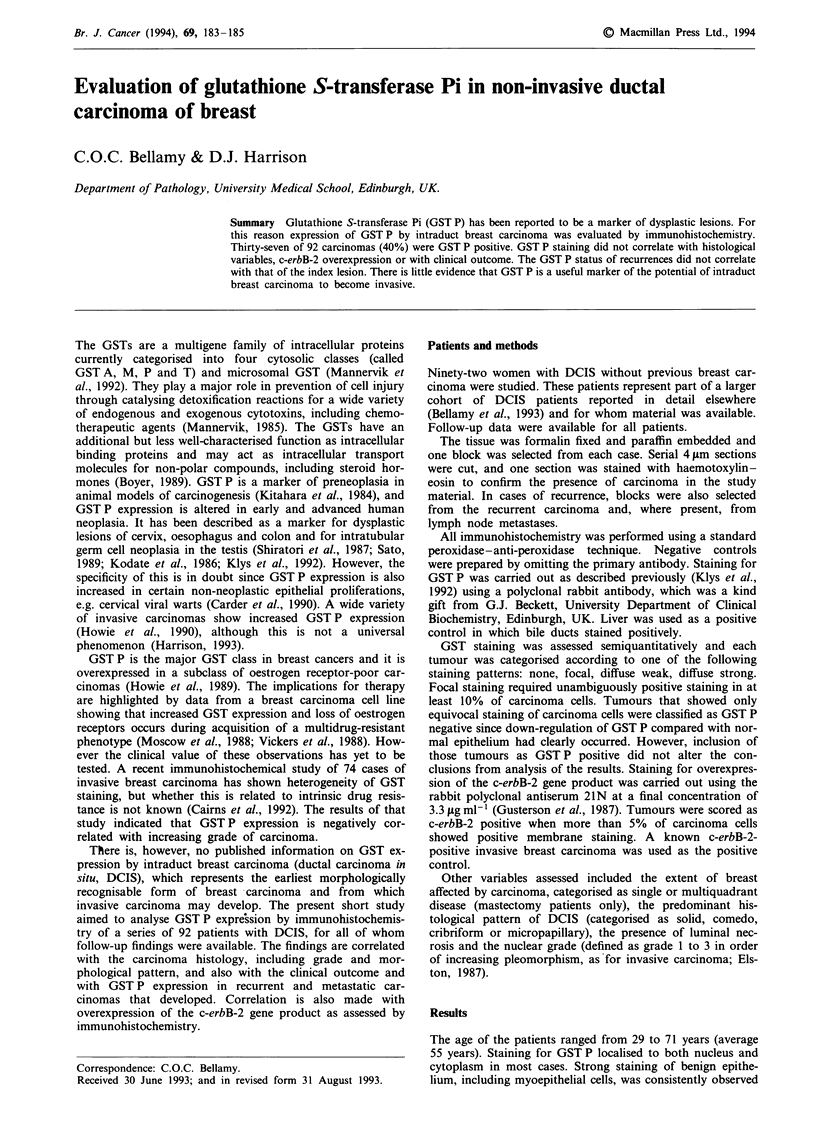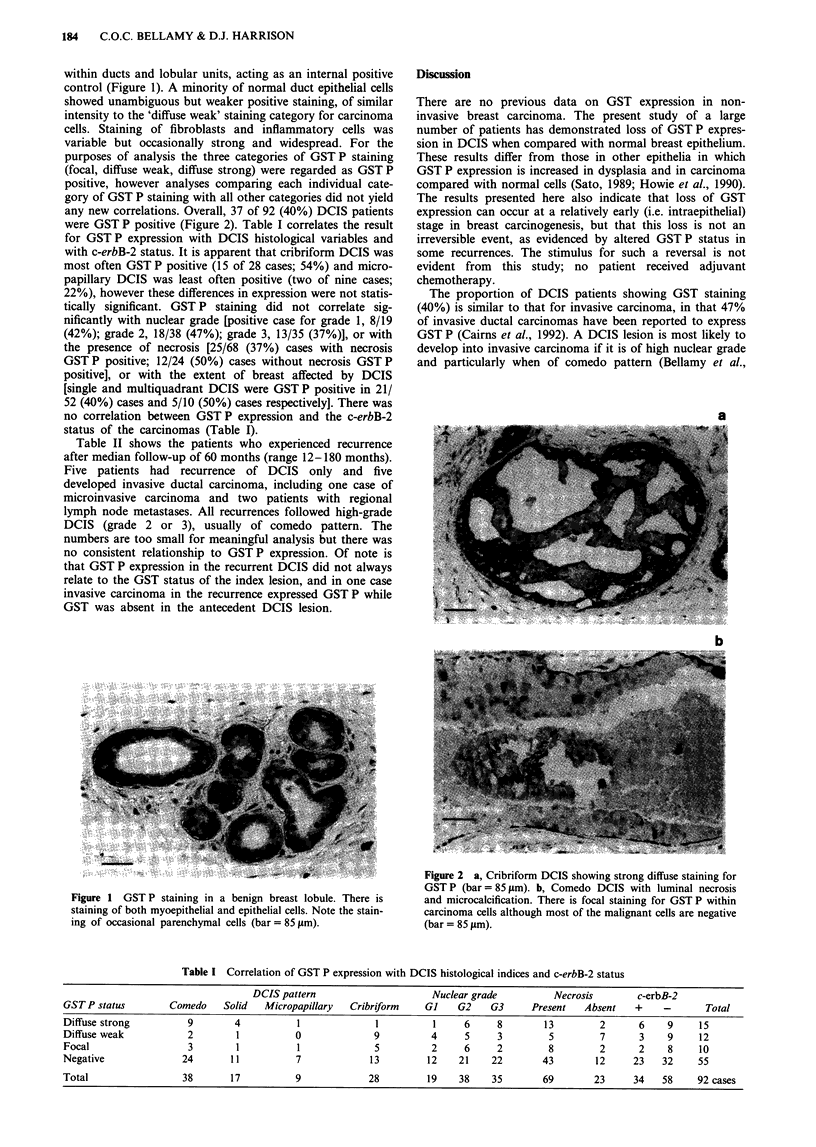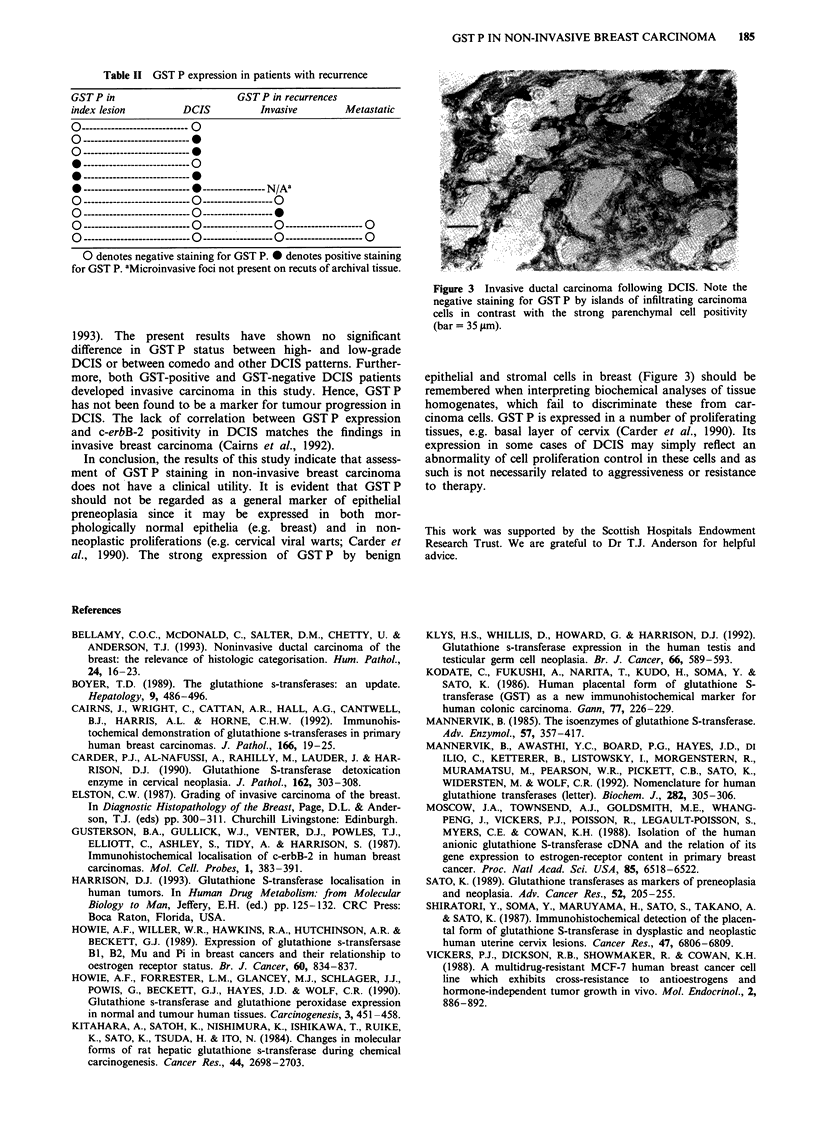# Evaluation of glutathione S-transferase Pi in non-invasive ductal carcinoma of breast.

**DOI:** 10.1038/bjc.1994.31

**Published:** 1994-01

**Authors:** C. O. Bellamy, D. J. Harrison

**Affiliations:** Department of Pathology, University Medical School, Edinburgh, UK.

## Abstract

**Images:**


Br. J. Cancer (1994), 69, 183–185 © Macmillan Press Ltd., 1994

### Evaluation of glutathione *S*-transferase Pi in non-invasive ductal carcinoma of breast

C.O.C. Bellamy & D.J. Harrison

*Department of Pathology, University Medical School, Edinburgh, UK.*

**Summary** Glutathione *S*-transferase Pi (GST P) has been reported to be a marker of dysplastic lesions. For this reason expression of GST P by intraduct breast carcinoma was evaluated by immunohistochemistry. Thirty-seven of 92 carcinomas (40%) were GST P positive. GST P staining did not correlate with histological variables, c-*erb*B-2 overexpression or with clinical outcome. The GST P status of recurrences did not correlate with that of the index lesion. There is little evidence that GST P is a useful marker of the potential of intraduct breast carcinoma to become invasive.

The GSTs are a multigene family of intracellular proteins currently categorised into four cytosolic classes (called GST A, M, P and T) and microsomal GST (Mannervik *et al.*, 1992). They play a major role in prevention of cell injury through catalysing detoxification reactions for a wide variety of endogenous and exogenous cytotoxins, including chemotherapeutic agents (Mannervik, 1985). The GSTs have an additional but less well-characterised function as intracellular binding proteins and may act as intracellular transport molecules for non-polar compounds, including steroid hormones (Boyer, 1989). GST P is a marker of preneoplasia in animal models of carcinogenesis (Kitahara *et al.*, 1984), and GST P expression is altered in early and advanced human neoplasia. It has been described as a marker for dysplastic lesions of cervix, oesophagus and colon and for intratubular germ cell neoplasia in the testis (Shiratori *et al.*, 1987; Sato, 1989; Kodate *et al.*, 1986; Klys *et al.*, 1992). However, the specificity of this is in doubt since GST P expression is also increased in certain non-neoplastic epithelial proliferations, e.g. cervical viral warts (Carder *et al.*, 1990). A wide variety of invasive carcinomas show increased GST P expression (Howie *et al.*, 1990), although this is not a universal phenomenon (Harrison, 1993).

GST P is the major GST class in breast cancers and it is overexpressed in a subclass of oestrogen receptor-poor carcinomas (Howie *et al.*, 1989). The implications for therapy are highlighted by data from a breast carcinoma cell line showing that increased GST expression and loss of oestrogen receptors occurs during acquisition of a multidrug-resistant phenotype (Moscow *et al.*, 1988; Vickers *et al.*, 1988). However the clinical value of these observations has yet to be tested. A recent immunohistochemical study of 74 cases of invasive breast carcinoma has shown heterogeneity of GST staining, but whether this is related to intrinsic drug resistance is not known (Cairns *et al.*, 1992). The results of that study indicated that GST P expression is negatively correlated with increasing grade of carcinoma.

There is, however, no published information on GST expression by intraduct breast carcinoma (ductal carcinoma *in situ*, DCIS), which represents the earliest morphologically recognisable form of breast carcinoma and from which invasive carcinoma may develop. The present short study aimed to analyse GST P expression by immunohistochemistry of a series of 92 patients with DCIS, for all of whom follow-up findings were available. The findings are correlated with the carcinoma histology, including grade and morphological pattern, and also with the clinical outcome and with GST P expression in recurrent and metastatic carcinomas that developed. Correlation is also made with overexpression of the c-*erb*B-2 gene product as assessed by immunohistochemistry.

Correspondence: C.O.C. Bellamy.
Received 30 June 1993; and in revised form 31 August 1993.

### Patients and methods

Ninety-two women with DCIS without previous breast carcinoma were studied. These patients represent part of a larger cohort of DCIS patients reported in detail elsewhere (Bellamy *et al.*, 1993) and for whom material was available. Follow-up data were available for all patients.

The tissue was formalin fixed and paraffin embedded and one block was selected from each case. Serial 4 μm sections were cut, and one section was stained with haemotoxylin–eosin to confirm the presence of carcinoma in the study material. In cases of recurrence, blocks were also selected from the recurrent carcinoma and, where present, from lymph node metastases.

All immunohistochemistry was performed using a standard peroxidase–anti-peroxidase technique. Negative controls were prepared by omitting the primary antibody. Staining for GST P was carried out as described previously (Klys *et al.*, 1992) using a polyclonal rabbit antibody, which was a kind gift from G.J. Beckett, University Department of Clinical Biochemistry, Edinburgh, UK. Liver was used as a positive control in which bile ducts stained positively.

GST staining was assessed semiquantitatively and each tumour was categorised according to one of the following staining patterns: none, focal, diffuse weak, diffuse strong. Focal staining required unambiguously positive staining in at least 10% of carcinoma cells. Tumours that showed only equivocal staining of carcinoma cells were classified as GST P negative since down-regulation of GST P compared with normal epithelium had clearly occurred. However, inclusion of those tumours as GST P positive did not alter the conclusions from analysis of the results. Staining for overexpression of the c-*erb*B-2 gene product was carried out using the rabbit polyclonal antiserum 21N at a final concentration of 3.3 $\mu g\ ml^{-1}$ (Gusterson *et al.*, 1987). Tumours were scored as c-*erb*B-2 positive when more than 5% of carcinoma cells showed positive membrane staining. A known c-*erb*B-2-positive invasive breast carcinoma was used as the positive control.

Other variables assessed included the extent of breast affected by carcinoma, categorised as single or multiquadrant disease (mastectomy patients only), the predominant histological pattern of DCIS (categorised as solid, comedo, cribriform or micropapillary), the presence of luminal necrosis and the nuclear grade (defined as grade 1 to 3 in order of increasing pleomorphism, as for invasive carcinoma; Elston, 1987).

### Results

The age of the patients ranged from 29 to 71 years (average 55 years). Staining for GST P localised to both nucleus and cytoplasm in most cases. Strong staining of benign epithelium, including myoepithelial cells, was consistently observed within ducts and lobular units, acting as an internal positive control (Figure 1). A minority of normal duct epithelial cells showed unambiguous but weaker positive staining, of similar intensity to the 'diffuse weak' staining category for carcinoma cells. Staining of fibroblasts and inflammatory cells was variable but occasionally strong and widespread. For the purposes of analysis the three categories of GST P staining (focal, diffuse weak, diffuse strong) were regarded as GST P positive, however analyses comparing each individual category of GST P staining with all other categories did not yield any new correlations. Overall, 37 of 92 (40%) DCIS patients were GST P positive (Figure 2). Table I correlates the result for GST P expression with DCIS histological variables and with c-*erb*B-2 status. It is apparent that cribriform DCIS was most often GST P positive (15 of 28 cases; 54%) and micropapillary DCIS was least often positive (two of nine cases; 22%), however these differences in expression were not statistically significant. GST P staining did not correlate significantly with nuclear grade [positive case for grade 1, 8/19 (42%); grade 2, 18/38 (47%); grade 3, 13/35 (37%)], or with the presence of necrosis [25/68 (37%) cases with necrosis GST P positive; 12/24 (50%) cases without necrosis GST P positive], or with the extent of breast affected by DCIS [single and multiquadrant DCIS were GST P positive in 21/52 (40%) cases and 5/10 (50%) cases respectively]. There was no correlation between GST P expression and the c-*erb*B-2 status of the carcinomas (Table I).

Table II shows the patients who experienced recurrence after median follow-up of 60 months (range 12–180 months). Five patients had recurrence of DCIS only and five developed invasive ductal carcinoma, including one case of microinvasive carcinoma and two patients with regional lymph node metastases. All recurrences followed high-grade DCIS (grade 2 or 3), usually of comedo pattern. The numbers are too small for meaningful analysis but there was no consistent relationship to GST P expression. Of note is that GST P expression in the recurrent DCIS did not always relate to the GST status of the index lesion, and in one case invasive carcinoma in the recurrence expressed GST P while GST was absent in the antecedent DCIS lesion.

**Figure 1** GST P staining in a benign breast lobule. There is staining of both myoepithelial and epithelial cells. Note the staining of occasional parenchymal cells (bar = 85 μm).

### Discussion

There are no previous data on GST expression in non-invasive breast carcinoma. The present study of a large number of patients has demonstrated loss of GST P expression in DCIS when compared with normal breast epithelium. These results differ from those in other epithelia in which GST P expression is increased in dysplasia and in carcinoma compared with normal cells (Sato, 1989; Howie *et al.*, 1990). The results presented here also indicate that loss of GST expression can occur at a relatively early (i.e. intraepithelial) stage in breast carcinogenesis, but that this loss is not an irreversible event, as evidenced by altered GST P status in some recurrences. The stimulus for such a reversal is not evident from this study; no patient received adjuvant chemotherapy.

The proportion of DCIS patients showing GST staining (40%) is similar to that for invasive carcinoma, in that 47% of invasive ductal carcinomas have been reported to express GST P (Cairns *et al.*, 1992). A DCIS lesion is most likely to develop into invasive carcinoma if it is of high nuclear grade and particularly when of comedo pattern (Bellamy *et al.*,

**Figure 2** **a**, Cribriform DCIS showing strong diffuse staining for GST P (bar = 85 μm). **b**, Comedo DCIS with luminal necrosis and microcalcification. There is focal staining for GST P within carcinoma cells although most of the malignant cells are negative (bar = 85 μm).

**Table I** Correlation of GST P expression with DCIS histological indices and c-*erb*B-2 status

| | DCIS pattern | | | | Nuclear grade | | | Necrosis | | c-erbB-2 | | |
|---|---|---|---|---|---|---|---|---|---|---|---|---|
| *GST P status* | *Comedo* | *Solid* | *Micropapillary* | *Cribriform* | *G1* | *G2* | *G3* | *Present* | *Absent* | + | − | *Total* |
| Diffuse strong | 9 | 4 | 1 | 1 | 1 | 6 | 8 | 13 | 2 | 6 | 9 | 15 |
| Diffuse weak | 2 | 1 | 0 | 9 | 4 | 5 | 3 | 5 | 7 | 3 | 9 | 12 |
| Focal | 3 | 1 | 1 | 5 | 2 | 6 | 2 | 8 | 2 | 2 | 8 | 10 |
| Negative | 24 | 11 | 7 | 13 | 12 | 21 | 22 | 43 | 12 | 23 | 32 | 55 |
| Total | 38 | 17 | 9 | 28 | 19 | 38 | 35 | 69 | 23 | 34 | 58 | 92 cases |

**Table II** GST P expression in patients with recurrence

| *GST P in index lesion* | *GST P in recurrences* DCIS | *Invasive* | *Metastatic* |
|---|---|---|---|
| ○ | ○ | | |
| ○ | ● | | |
| ○ | ● | | |
| ● | ○ | | |
| ● | ● | | |
| ● | ● | N/A[a] | |
| ○ | ○ | ○ | |
| ○ | ○ | ● | |
| ○ | ○ | ○ | ○ |
| ○ | ○ | ○ | ○ |

○ denotes negative staining for GST P. ● denotes positive staining for GST P. [a]Microinvasive foci not present on recuts of archival tissue.

**Figure 3** Invasive ductal carcinoma following DCIS. Note the negative staining for GST P by islands of infiltrating carcinoma cells in contrast with the strong parenchymal cell positivity (bar = 35 μm).

1993). The present results have shown no significant difference in GST P status between high- and low-grade DCIS or between comedo and other DCIS patterns. Furthermore, both GST-positive and GST-negative DCIS patients developed invasive carcinoma in this study. Hence, GST P has not been found to be a marker for tumour progression in DCIS. The lack of correlation between GST P expression and c-*erb*B-2 positivity in DCIS matches the findings in invasive breast carcinoma (Cairns *et al*., 1992).

In conclusion, the results of this study indicate that assessment of GST P staining in non-invasive breast carcinoma does not have a clinical utility. It is evident that GST P should not be regarded as a general marker of epithelial preneoplasia since it may be expressed in both morphologically normal epithelia (e.g. breast) and in non-neoplastic proliferations (e.g. cervical viral warts; Carder *et al*., 1990). The strong expression of GST P by benign epithelial and stromal cells in breast (Figure 3) should be remembered when interpreting biochemical analyses of tissue homogenates, which fail to discriminate these from carcinoma cells. GST P is expressed in a number of proliferating tissues, e.g. basal layer of cervix (Carder *et al*., 1990). Its expression in some cases of DCIS may simply reflect an abnormality of cell proliferation control in these cells and as such is not necessarily related to aggressiveness or resistance to therapy.

This work was supported by the Scottish Hospitals Endowment Research Trust. We are grateful to Dr T.J. Anderson for helpful advice.

### References

BELLAMY, C.O.C., McDONALD, C., SALTER, D.M., CHETTY, U. & ANDERSON, T.J. (1993). Noninvasive ductal carcinoma of the breast: the relevance of histologic categorisation. *Hum. Pathol.*, **24**, 16–23.

BOYER, T.D. (1989). The glutathione s-transferases: an update. *Hepatology*, **9**, 486–496.

CAIRNS, J., WRIGHT, C., CATTAN, A.R., HALL, A.G., CANTWELL, B.J., HARRIS, A.L. & HORNE, C.H.W. (1992). Immunohistochemical demonstration of glutathione s-transferases in primary human breast carcinomas. *J. Pathol.*, **166**, 19–25.

CARDER, P.J., AL-NAFUSSI, A., RAHILLY, M., LAUDER, J. & HARRISON, D.J. (1990). Glutathione S-transferase detoxication enzyme in cervical neoplasia. *J. Pathol.*, **162**, 303–308.

ELSTON, C.W. (1987). Grading of invasive carcinoma of the breast. In *Diagnostic Histopathology of the Breast*, Page, D.L. & Anderson, T.J. (eds) pp. 300–311. Churchill Livingstone: Edinburgh.

GUSTERSON, B.A., GULLICK, W.J., VENTER, D.J., POWLES, T.J., ELLIOTT, C., ASHLEY, S., TIDY, A. & HARRISON, S. (1987). Immunohistochemical localisation of c-erbB-2 in human breast carcinomas. *Mol. Cell. Probes*, **1**, 383–391.

HARRISON, D.J. (1993). Glutathione S-transferase localisation in human tumors. In *Human Drug Metabolism: from Molecular Biology to Man*, Jeffery, E.H. (ed.) pp. 125–132. CRC Press: Boca Raton, Florida, USA.

HOWIE, A.F., WILLER, W.R., HAWKINS, R.A., HUTCHINSON, A.R. & BECKETT, G.J. (1989). Expression of glutathione s-transferase B1, B2, Mu and Pi in breast cancers and their relationship to oestrogen receptor status. *Br. J. Cancer*, **60**, 834–837.

HOWIE, A.F., FORRESTER, L.M., GLANCEY, M.J., SCHLAGER, J.J., POWIS, G., BECKETT, G.J., HAYES, J.D. & WOLF, C.R. (1990). Glutathione s-transferase and glutathione peroxidase expression in normal and tumour human tissues. *Carcinogenesis*, **3**, 451–458.

KITAHARA, A., SATOH, K., NISHIMURA, K., ISHIKAWA, T., RUIKE, K., SATO, K., TSUDA, H. & ITO, N. (1984). Changes in molecular forms of rat hepatic glutathione s-transferase during chemical carcinogenesis. *Cancer Res.*, **44**, 2698–2703.

KLYS, H.S., WHILLIS, D., HOWARD, G. & HARRISON, D.J. (1992). Glutathione s-transferase expression in the human testis and testicular germ cell neoplasia. *Br. J. Cancer*, **66**, 589–593.

KODATE, C., FUKUSHI, A., NARITA, T., KUDO, H., SOMA, Y. & SATO, K. (1986). Human placental form of glutathione S-transferase (GST) as a new immunohistochemical marker for human colonic carcinoma. *Gann*, **77**, 226–229.

MANNERVIK, B. (1985). The isoenzymes of glutathione S-transferase. *Adv. Enzymol.*, **57**, 357–417.

MANNERVIK, B., AWASTHI, Y.C., BOARD, P.G., HAYES, J.D., DI ILIO, C., KETTERER, B., LISTOWSKY, I., MORGENSTERN, R., MURAMATSU, M., PEARSON, W.R., PICKETT, C.B., SATO, K., WIDERSTEN, M. & WOLF, C.R. (1992). Nomenclature for human glutathione transferases (letter). *Biochem. J.*, **282**, 305–306.

MOSCOW, J.A., TOWNSEND, A.J., GOLDSMITH, M.E., WHANG-PENG, J., VICKERS, P.J., POISSON, R., LEGAULT-POISSON, S., MYERS, C.E. & COWAN, K.H. (1988). Isolation of the human anionic glutathione S-transferase cDNA and the relation of its gene expression to estrogen-receptor content in primary breast cancer. *Proc. Natl Acad. Sci. USA*, **85**, 6518–6522.

SATO, K. (1989). Glutathione transferases as markers of preneoplasia and neoplasia. *Adv. Cancer Res.*, **52**, 205–255.

SHIRATORI, Y., SOMA, Y., MARUYAMA, H., SATO, S., TAKANO, A. & SATO, K. (1987). Immunohistochemical detection of the placental form of glutathione S-transferase in dysplastic and neoplastic human uterine cervix lesions. *Cancer Res.*, **47**, 6806–6809.

VICKERS, P.J., DICKSON, R.B., SHOWMAKER, R. & COWAN, K.H. (1988). A multidrug-resistant MCF-7 human breast cancer cell line which exhibits cross-resistance to antioestrogens and hormone-independent tumor growth in vivo. *Mol. Endocrinol.*, **2**, 886–892.